# A *tetra*-*ortho*-Chlorinated Azobenzene Molecule for Visible-Light Photon Energy Conversion and Storage

**DOI:** 10.3390/molecules30112333

**Published:** 2025-05-27

**Authors:** Shuxin Tang, Yating Zhang, Jun Xia, Jing Qi, Fan Tang, Fei Zhai, Liqi Dong

**Affiliations:** 1 Key Laboratory of Pollution Exposure and Health Intervention of Zhejiang Province, Interdisciplinary Research Academy, Zhejiang Shuren University, Hangzhou 310021, China; 18267180254@163.com (S.T.);; 2 College of Environment, Zhejiang University of Technology, 18 Chaowang RD, Hangzhou 310014, China; 3 Shandong Laboratory of Advanced Materials and Green Manufacturing at Yantai, Yantai Zhongke Research Institute of Advanced Materials and Green Chemical Engineering, Yantai 264006, China; 4 Zhejiang Collaborative Innovation Center for Full-Process Monitoring and Green Governance of Emerging Contaminants, Hangzhou 310021, China

**Keywords:** azobenzene, photothermal, visible-light energy storage, photoinduced phase transition

## Abstract

The development of photoactive molecules for photothermal energy storage is a focus of research in solar energy utilization technology. Azobenzene photoswitch has emerged as a promising candidate for solar energy conversion and storage due to its unique photoisomerization characteristics. Nonetheless, a majority of azobenzene-based molecular photothermal systems have a significant drawback: they depend on ultraviolet light for *E*-to-*Z* isomerization to store photon energy rather than visible light, which seriously hinders the development of azobenzene photoswitch in practical solar energy utilization applications. In this study, an azobenzene photothermal molecule that can effectively store visible-light photon energy was design and synthesized, which includes a *tetra*-*ortho*-chlorinated azo structure as the “head” part and an alkyl chain at *para*-position as the “tail” part. The ultraviolet–visible and ^1^H NMR spectrum indicated that the obtained *tetra*-*ortho*-chlorinated azobenzene photothermal molecule could effectively absorb and store photon energy under 550 nm irradiation and release the stored energy upon 430 nm light irradiation. The storage energy density of the charged azobenzene photothermal molecule was determined to be 13.50 kJ/mol through differential scanning calorimetry and 28.21 kJ/mol via density functional theory theoretical calculations. This discrepancy was ascribed to the 64% Z-isomer yield harvesting during the charging process. Furthermore, the obtained *tetra*-*ortho*-chlorinated azobenzene exhibited long-term energy storage (approximately 11 days of half-life) and cyclic stability (100 cycles). Notably, the *E*-isomer of *tetra*-*ortho*-chlorinated azobenzene exhibited a high degree of supercooling, which may be advantageous for use in extremely low-temperature environments.

## 1. Introduction

Azobenzene-based molecular photothermal systems, also known as molecular solar thermal systems, offer a promising approach to solar energy conversion and storage, attributed to their distinctive photoisomerization characteristics [1,2]. Azobenzene photoswitch facilitates dynamic energy conversion and storage through *trans*-to-*cis* (*E*-to-*Z*) molecular configuration transitions. This process effectively captures photon energy, converting it into chemical bond energy for storage, which can be subsequently released as thermal energy on demand [3]. This innovative photo energy utilization strategy transcends traditional energy conversion methods, such as direct light-to-heat conversion, electricity-mediated thermal generation, and phase-change latent heat systems, significantly enhancing the efficiency of photo energy utilization [4]. This photothermal system boasts remarkable advantages, including closed-cycle operation, zero emissions, safety, cleanliness, and ease of transportation, establishing its potential for diverse applications encompassing personal thermal management [5,6,7,8], solar-thermal textiles [9,10,11,12], and anti-/de-icing technologies [13,14].

However, a critical limitation faced by current azobenzene-based molecular photothermal energy storage technology is that the *E*-to-*Z* switching usually requires ultraviolet irradiation (typically 365 nm), which comprises only 5% of solar energy and has limited penetration capabilities [15]. Furthermore, prolonged exposure to ultraviolet light may also cause material degradation, severely limiting the practical application of azobenzene in photothermal materials. In contrast, visible light, which constitutes approximately 50% of the solar spectrum and is the primary component of sunlight, presents an opportunity for enhanced solar energy utilization. An optimal azobenzene-based molecular photothermal system should effectively absorb a substantial portion of solar photons, especially from the intense visible-light region of the solar spectrum. Nevertheless, visible light generally triggers the *Z*-to-*E* isomerization of azobenzene molecules, predominantly serving as a stimulus for the heat release during the discharge process [16,17,18]. This poses a challenge in the design of an azobenzene photoswitch that can effectively store visible-light photon energy.

To address this challenge, scientists have proposed two primary strategies. The first strategy involves the integration of upconversion materials, which convert long-wavelength radiation from sunlight into short-wavelength radiation (such as UV light) that can subsequently be absorbed by azobenzene molecules [19,20,21]. The Moth–Poulsen research group demonstrated the first example of a far-red (740 nm) triplet sensitized *Z*-to-*E* photoswitching of azobenzene in a condensed phase through the co-assembly of a liquid azobenzene derivative with a liquid surfactant–protein film [22]. The novel bioplastics technology offers a sustainable platform for the development of solid-state photoswitching materials for energy harvesting. Yu et al. incorporated upconversion nanophosphors into a cross-linked liquid–crystal polymer film with azotolane, enabling rapid bending under continuous-wave near-infrared light at 980 nm [23]. However, the conversion efficiency of upconversion materials is often constrained by their low luminescence efficiency and complex preparation processes. The second strategy, which is the primary focus of the current research, aims to create azobenzene structures capable of efficiently storing photon energy from visible light through molecular design. This method focuses on adding specific substituents or modifying the molecular backbone to either separate the n–π* bands within the visible spectrum or shift the π–π* transition to longer wavelengths. Herges et al. [24,25] and Woolley et al. [26] synthesized ethylene-bridged and *tetra*-*ortho*-methoxy azobenzene derivatives to separate the n–π* transitions of the *Z* and *E* isomers. The distinct n–π* absorption bands in these azobenzene derivatives allow for *E*-to-*Z* and *Z*-to-*E* photoisomerization when exposed to visible light. Recent studies have shown that *tetra*-*ortho*-halogenate azobenzene derivatives exhibit significant potential for visible-light photon energy storage due to their capacity to induce a substantial red-shift in the *E*-isomer’s absorption spectrum, making them responsive to visible light [5,17,27,28,29,30,31]. For example, the *Z*-isomer of the *tetra*-*ortho*-fluorinated azobenzene exhibits a thermal half-life of 700 days and can be photoisomerized using green light for *E*-to-*Z* conversion and blue light for *Z*-to-*E* reversion [28]. In addition, several azopyrazole derivatives also show efficient *E*-to-*Z* and *Z*-to-*E* isomerization under 405 and 520/532 nm light irradiations, respectively [6,32,33]. In summary, molecular design engineering provides an innovative, efficient, and versatile method for the fabrication of azobenzene-based molecular photothermal storage system that effectively store visible-light photon energy.

We designed and synthesized an azobenzene photothermal molecule that stores visible-light photon energy through molecular engineering. The molecule consists of a *tetra*-*ortho*-chlorinated azo structure as the “head” part and an alkyl chain at *para*-position as the “tail” part. By grafting chlorine atoms on the *ortho* positions of azobenzene molecule, we red-shifted the π–π* transition to the green portion (520–550 nm) of the solar spectrum. The photoisomerization of *tetra*-*ortho*-chlorinated azobenzene was examined using ultraviolet–visible (UV-vis) spectroscopy, revealing that *E*-to-*Z* isomerization occurs under 550 nm light irradiation, demonstrating the molecule’s ability to absorb and store green photon energy. The *Z*-to-*E* isomerization under 430 nm light irradiation indicates that the molecule can release stored energy when exposed to blue light. In addition, the energy storage density, energy storage lifetime, and cyclic stability of the obtained azobenzene molecule were systematically evaluated via UV–vis analysis, differential scanning calorimetry (DSC) measurement, and theoretical DFT calculation, resulting in an energy density of 13.50 kJ/mol, half-life of 10.97 days, and charging–discharging cycling performance of 100 times. It is worth mentioning that the alkyl chain at the *para*-position endows the molecule with a controlled optical crystal-to-liquid phase transition and high supercooling performance, which may be beneficial for the charging process and its application in extremely low-temperature environments. This study adds a new member to the family of azobenzene-based photothermal storage materials capable of efficiently storing visible-light photon energy, further advancing this field towards practical applications.

## 2. Results and Discussion

### 2.1. Molecule Design and Synthesis

**Scheme 1:**
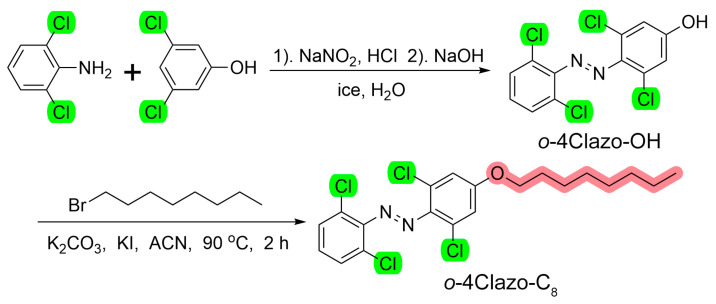
Synthetic route for the *o*-4ClAzo-C_8_ compound.

In recent years, it has been demonstrated that *tetra*-*ortho*-halogenate azobenzene exhibits a remarkable capacity to separate n→π* transitions, which allows for the generation of *Z*-isomers via irradiation with yellow or red light [17,27,29,34]. Therefore, *tetra*-*ortho*-chlorinated azobenzene was chosen as the photoswitching structure, and an alkyl chain was incorporated in *para*-positions to endow the azobenzene molecules with the photoinduced phase transition feature. As the photoinduced *Z*-liquid can facilitate a decline in the melting point of the *E*/*Z* eutectic mixture, the liquid-state *Z*-isomer will play the role of a “solvent” that supplies free volume and thereby allows for further *E*-crystal-to-*Z*-rich liquid photoisomerization [35,36,37,38,39,40]. The target product *o*-4ClAzo-C_8_ was obtained through the azo-coupling between substituted phenols and anilines [41], followed by the Williamson ether reaction [5,15]. The synthesis route is shown in Scheme 1. The characterization data, including the ^1^H and ^13^C NMR spectra of products, are included in the Supplementary Materials.

### 2.2. Light Source Optimization

**Figure 1:**
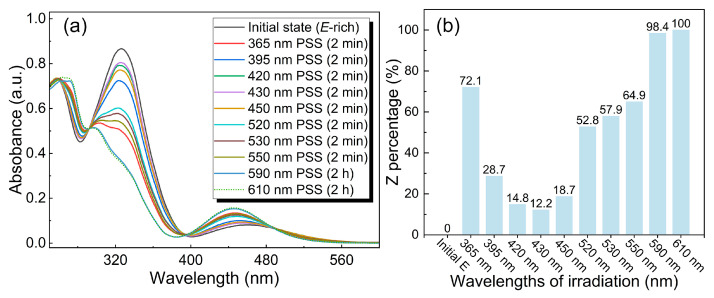
(**a**) UV–vis absorption spectra of the *o*-4ClAzo-C_8_ molecule in dichloromethane (DCM) solution (1 × 10^−4^ mol L^−1^), irradiated with difference wavelengths of light sources. (**b**) The percentage content of the *Z* isomer in the *o*-4ClAzo-C_8_ molecule at PSS under different wavelengths of light irradiation.

We systematically investigated the photostationary state (PSS) behavior of the *o*-4ClAzo-C_8_ compound under various wavelengths of light by conducting UV–vis spectroscopic measurements (Figure 1a). Subsequently, the percentages of *Z*-isomers in the resulting photostationary states (PSS) were calculated using Equation (S1) (Figure 1b). As illustrated in Figure 1a, the initial *E*-rich state of *o*-4ClAzo-C_8_ compound exhibits two distinct absorption peaks: a π–π* transition peak at 326 nm and an n–π* transition peak at 457 nm. Upon conversion to the Z-rich state, the absorption intensity at 326 nm decreased significantly, and the n–π* transition peak exhibited a slight blue shift accompanied by a slight increase in intensity. The light sources at 365 nm, 520 nm, 530 nm, 550 nm, 590 nm, and 610 nm induced the *E*-to-*Z* photoisomerization of the *o*-4ClAzo-C_8_, corresponding to the charging process of the photothermal energy storage materials. In the visible-light range, despite the fact that orange light (590 nm and 610 nm) irradiation resulted in a PSS containing almost only the *Z* isomer, these wavelengths required more time (2 h) for photoisomerization. The 550 nm light-induced PSS could harvest a 64.9% Z-isomer, slightly less than that of 365 nm light irradiation (72.1%). Consequently, 550 nm was identified as the optimal visible-light source for the charging of *o*-4ClAzo-C_8_ based photothermal energy storage materials. Conversely, light sources at 395 nm, 420 nm, 430 nm, and 450 nm triggered the *Z*-to-*E* reverse isomerization corresponding to the discharging process. In the visible-light range, the PSS induced by blue light (430 nm) demonstrated the lowest *Z*-isomer content (12.2%), indicating that blue light can be effectively employed for back-isomerization (Figure 1b). Therefore, to release heat from the charged *o*-4ClAzo-C_8_ molecule, irradiation with 430 nm light is recommended.

### 2.3. Photoisomerization Performance

**Figure 2:**
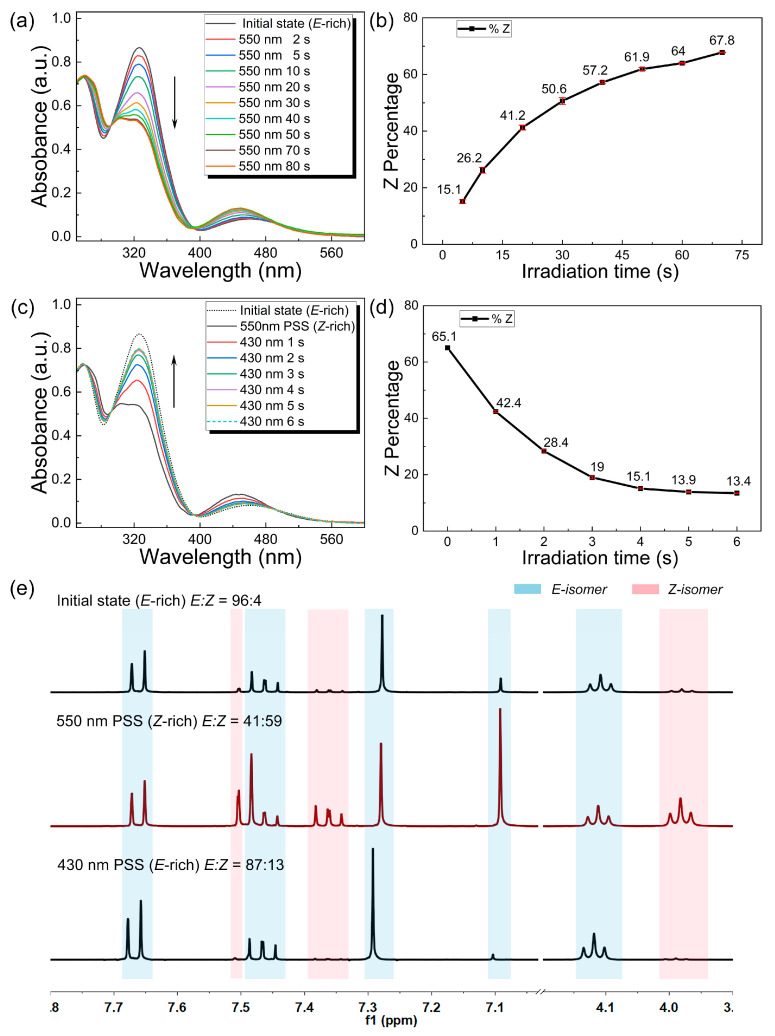
Time-evolved UV–vis spectra of *o*-4ClAzo-C_8_ samples under irradiations of 550 nm (**a**) and 430 nm (**c**). The spectra were normalized with respect to the isosbestic point at 332 nm. The arrows indicate the order of tests performed. Z-isomer percentage of *o*-4ClAzo-C_8_ samples vs. 550 nm irradiation time (**b**) and 430 nm irradiation time (**d**), respectively. (The numerical values were calculated using Equation (S1).) (**e**) The ^1^H NMR spectrum and isomeric composition of the initial *o*-4ClAzo-C_8_ (top) after charging under 550 nm light (middle) and discharging under 430 nm light (bottom).

The photoisomerization of the *o*-4ClAzo-C_8_ molecule under 550 nm and 430 nm light irradiation was studied using UV–vis absorption spectra at room temperature (Figure 2a,c). As the irradiation time increased during the charging, the π–π* transition peak intensity significantly decreased, while the n–π* transition peak slightly increased and exhibited a blue shift from 457 nm to 447 nm, indicating isomerization from *E* to *Z* isomers (Figure 2a). This time-evolution ultimately led to the attainment of the *Z*-rich photostationary. During the discharging process, the π–π* transition peak increased, while the n–π* transition peak decreased due to the *Z*-to-*E* reversion (Figure 2c). To further investigate the efficiency of the charging and discharging processes, we monitored the isomeric compositions at various durations of light irradiation. The irradiated samples were characterized using UV–vis and ^1^H NMR spectroscopy. In the initial state, the *E*/*Z* mixture of *o*-4ClAzo-C_8_ compound was primarily composed of the *E*-isomer (*E*:*Z* = 96:4, calculated using Equation (S2)), as determined from the NMR spectrum (Figure 2e, top). During the photo-charging process, the sample was irradiated with 550 nm visible light, resulting in a fraction of 67.8% of the *Z* isomer within 70 s (Figure 2a,b). It is noteworthy that the *Z*-fraction calculated from the UV–vis spectroscopy data was slightly higher than that derived from the ^1^H NMR spectra (*E*:*Z* = 41:59) (Figure 2e, middle). This discrepancy may be attributable to the inherent error om the UV–vis spectroscopic data calculation method. In the photo-discharging process, the *Z* isomer content dropped to 13.4% after 6 s of 430 nm light illumination (Figure 2c,d), consistent with the NMR results (*E*:*Z* = 87:13) (Figure 2e, bottom). The rapid discharging process (6 s) under 430 nm blue light indicated the potential for rapid heat release within a short timeframe. Based on the above analysis, its efficient bidirectional photoisomerization, with excellent reversibility of *o*-4ClAzo-C_8_, renders it a promising candidate for energy harvesting applications.

### 2.4. Energy Storage Lifetime 

**Figure 3:**
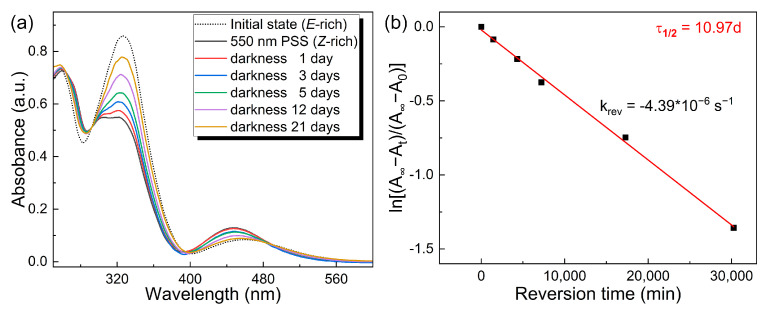
(**a**) The UV–vis absorption spectra of the *o*-4ClAzo-C_8_ sample, which was charged with 550 nm light and naturally recovered in the dark at room temperature over time. (**b**) The first-order rate constants for *Z*-to-*E* reversion (k_rev_) in darkness for *o*-4ClAzo-C_8_.

In addition to being triggered by blue light, the *Z*-rich sample exhibits spontaneous reversion back to the *E*-rich state in darkness. This dynamic thermal relaxation behavior provides insights into the isomerization energy storage time of the *o*-4ClAzo-C_8_ compound, which is crucial to improve the energy storage lifetime and controllable energy release. Figure 3a illustrates a continuous increase in the peak intensity of the π–π* transition for the 550 nm PSS sample over time, indicating that the *Z*-isomer in the molecule gradually reverts to the *E* form. Until day 21, the *E*-isomer content in the *E*/*Z* mixture of the *o*-4ClAzo-C_8_ compound reaches approximately 83% (calculated by Equation (S1)). This observation indicates that the thermal *Z*-to-*E* reversion occurs very slowly in darkness, which is beneficial to control the heat release of *o*-4ClAzo-C_8_-based molecular photothermal systems. According to Equation (S3), the first-order rate of the *Z*-to-*E* isomerization (4.39 × 10^−6^ s^−1^) is four orders of magnitude lower than that of pure azobenzene (4.15 × 10^−2^ s^−1^) [42]. The calculated half-life of the *Z* isomer is 10.97 days (Equation (S4)), more than twice that of the pure azobenzene molecule (Figure 3b) [43]. This result suggests that the *o*-4ClAzo-C_8_ photothermal molecule could be utilized in long-term azobenzene-based photothermal systems. In addition, the synergistic effect of chlorine’s steric distortion and the alkoxy group’s electronic modulation likely amplifies the kinetic instability of the *Z*-isomer compared to the pristine *tetra*-*ortho*-fluorinated azobenzene fluorinated analogs [28], and thereby significantly decrease the half-life of the *Z*-isomer for the *o*-4ClAzo-C_8_ compound.

**Figure 4:**
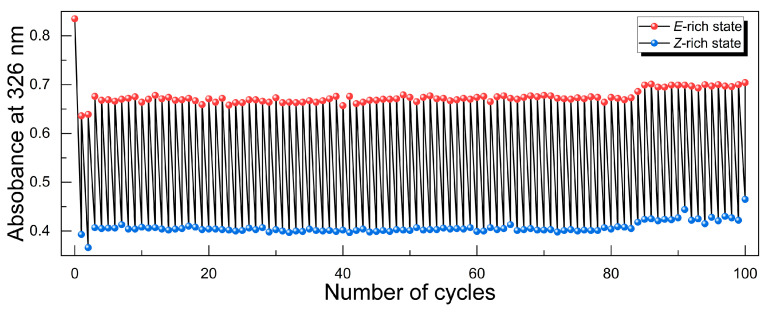
The stability of cycling through alternating charging and discharging over 100 cycles.

We investigated the cycling stability of energy charging and heat releasing in the *o*-4ClAzo-C_8_ molecule through repeated *E*-to-*Z* photoisomerization (charging process) and *Z*-to-*E* photoreversion (discharging processes). The initial sample was charged to its PSS (i.e., *Z*-rich state) under 550 nm light irradiation, and then discharged back to the *E*-rich state with 430 nm light exposure. Figure 4 depicts the UV–vis spectroscopy-recorded absorbance changes in the π–π* transition peak for *E*-rich and *Z*-rich states after 550 or 430 nm alternating-light irradiations. The results demonstrate that the *o*-4ClAzo-C_8_ photothermal molecule exhibits favorable cycling stability over 100 complete cycles without notable photodegradation.

### 2.5. Phase Transition Property

**Figure 5:**
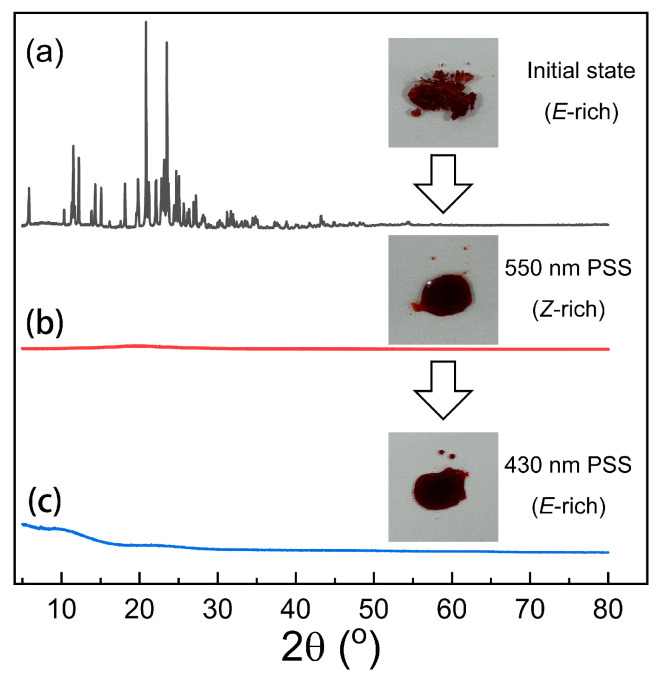
X-ray diffraction patterns of *o*-4ClAzo-C_8_ molecule: (**a**) initial state sample, (**b**) after 550 nm irradiation, and (**c**) after 430 nm irradiation. A digital photograph of each sample is shown as an inset.

XRD analysis was performed to examine the phase transition of *o*-4ClAzo-C_8_ from an *E*-crystal to *Z*-liquid. As shown in Figure 5a, the initial state featured sharp peaks at 2θ values ranging from 5° to 25°, which correspond to the ordered stacking of the *E*-crystal. This XRD pattern is characteristic of the *E* configuration in azobenzene compounds [13,44,45]. Subsequently, these characteristic peaks vanished upon irradiation with 550 nm light, indicating that the *Z*-isomer was in an amorphous state, accompanied by the photo-melting of the *E*-crystal into a *Z*-liquid (Figure 5b inset). These observations clearly demonstrate that the *E*-to-*Z* photoisomerization of *o*-4ClAzo-C_8_ leads to its liquefaction into an isotropic liquid phase. However, the subsequent irradiation of the *Z*-liquid with 430 nm light did not cause the *E*-crystal to reappear, but instead produced *E*-liquid (Figure 5c), with no recovery of the initial XRD pattern. This intriguing result may be attributed to four factors: (1) the *tetra*-*ortho*-substituted chlorine atom likely impedes the ordered molecular arrangement through steric hindrance, which significantly increasing the difficulty of molecular packing and thereby reduces the crystallization ability of the *Z*-liquid [29]; (2) the introduction of the *para*-alkyl chain drastically alters the molecular symmetry, disrupting the π–π stacking of Azobenzene molecules and making it difficult for them to reorganize into ordered crystals after melting [13]; (3) the conjugated double bonds in azobenzene and the electron-withdrawing effect of the chlorine atom may enhance molecular rigidity, inhibiting molecular chain mobility and obstructing the formation of crystal nuclei [17,45]; (4) the liquid state of the *E*-isomer possesses a relatively high degree of supercooling.

### 2.6. Energy Storage Density

**Figure 6:**
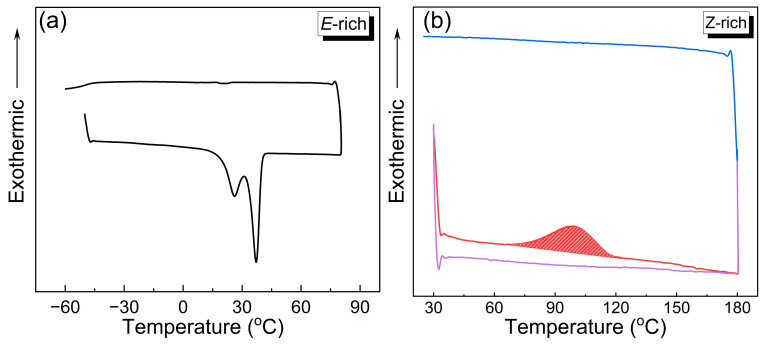
DSC curves of *o*-4ClAzo-C_8_ for (**a**) initial state (*E*-rich) and (**b**) after charging under 550 nm light for 30 min (*Z*-rich), including heating (red), cooling (blue), and reheating (purple) segments.

The energy storage capacity of the *o*-4ClAzo-C_8_ photothermal molecule was thoroughly investigated. Figure 6a presents the DSC thermograms for the *E*-rich. The DSC trace of the *E*-isomer showcases a distinct endothermic melting peak at approximately 37 °C during the heating phase, indicative of a phase transition from a crystalline to a liquid state. Notably, the absence of a detectable exothermic crystallization peak during the cooling phase (temperature range: 80 °C to −60 °C) emphasizes the pronounced supercooling exhibited by the *E*-isomer of *o*-4ClAzo-C_8_ (consistent with previous XRD observations). In the subsequent reheating cycle, no endothermic melting peak in the first heating cycle was observed again (Figure S7), further confirming the *E*-configuration can maintain its liquid state across a wider temperature range, thereby effectively preserving the phase change’s latent heat. This distinctive thermal behavior could position the *o*-4ClAzo-C_8_ photothermal molecule as a promising candidate for advanced heat-preservation applications in extreme low-temperature environments.

**Figure 7:**
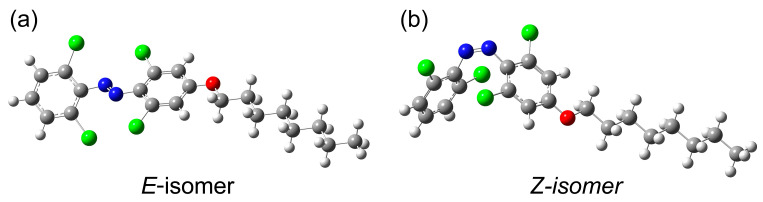
The optimized geometric structure of *o*-4ClAzo-C_8_ with the *E*-isomer (**a**) and *Z*-isomer (**b**). Green, blue and red represent Cl, N, and O, respectively.

Figure 6b presents the DSC thermograms of *Z*-rich *o*-4ClAzo-C_8_ photothermal molecules. In the heating stage, a broad exothermic peak was observed within the temperature range of 70 °C to 120 °C, with a ΔH value of 30.2 J/g (13.5 kJ/mol). The observed peak corresponds to thermal *Z*-to-*E* isomerization, which disappears during the reheating phase. This suggests that the exothermic heat flow observed in the first heating cycle originates from photo-induced energy storage. Due to the significant supercooling of the *E*-liquid, no crystallization peak was observed during the cooling phase either. Theoretical calculations indicated an energy gap of 28.21 kJ/mol between the *Z*-isomer and *E*-isomer (Figure 7a,b), which is twice the observed experimental value. This discrepancy is associated with the low degree of *E*-to-*Z* isomerization during the charging process (with only 64% of the *Z*-isomer yield achieved). Compared with the reported azobenzene photothermal molecule of the same type [17,30,34,38], this work has a relatively moderate energy density value. Although the *Z*-isomer shows a relatively moderate energy density, the significant supercooling of the *E*-isomer presents a promising opportunity for its use in temperature buffering systems in extreme low-temperature environments; it could potentially provide antifreeze protection for equipment used in polar or space exploration and future protective clothing.

## 3. Materials and Methods

### 3.1. Materials

In this study, 3,5-dichlorophenol (98%), 2,6-dichloroaniline (99%), sodium hydroxide (NaOH, 98%), sodium nitrite (NaNO_2_, 99%), 1-bromooctane (99%), potassium iodide (KI, 99.8%), and potassium carbonate (K_2_CO_3_, 99.9%) were purchased from Aladdin Biochemical Technology Co., Ltd. (Shanghai, China). Hexyl hydride (AR), ethyl acetate (AR), dichloromethane (DCM, AR and SP), acetonitrile (ACN, AR), and hydrochloric acid (HCl, AR) were purchased from Titan Scientific Co., Ltd. (Shanghai, China). All the reagents were used directly without further purification.

### 3.2. Synthesis

#### 3.2.1. Synthesis of (E)-3,5-Dichloro-4-((2,6-dichlorophenyl)diazenyl)phenol (*o*-4ClAzo-OH)

A solution of 2,6-dichloroaniline (648 mg, 4 mmol) in 10 mL of distilled water was acidified with 1 mL of concentrated hydrochloric acid and cooled to 0 °C. A cold solution of sodium nitrite (304 mg, 4.4 mmol) was added dropwise to 5 mL distilled water. The orange suspension was then added dropwise into a chilled solution of 3,5-dichlorophenol (717 mg, 4.4 mmol) and sodium hydroxide (1.5 M, 8 mL). The mixture was stirred at 0 °C for 2 h before being acidified to pH 2 using 0.5 M hydrochloric acid. The product underwent extraction with DCM three times, followed by washing the organic layer with saturated sodium chloride solution. It was then dried using anhydrous sodium sulfate and concentrated using a rotary evaporator. Purification via flash silica gel chromatography (hexyl hydride–ethyl acetate = 7:2) yielded the desired orange solid product (590 mg, 44%). The ^1^H and ^13^C NMR spectrum are displayed in the Supporting Materials (Figures S1 and S2).

#### 3.2.2. Synthesis of (E)-1-(2,6-Dichloro-4-(octyloxy)phenyl)-2-(2,6-dichlorophenyl)diazene (*o*-4ClAzo-C_8_)

*o*-4Clazo-OH (672 mg, 2 mmol), potassium iodide (664 mg, 4 mmol), and potassium carbonate (553 mg, 4 mmol) were dissolved in 10 mL acetonitrile, followed by the dropwise addition of 1-bromooctane (0.418 mL, 2.4 mmol). The mixture was stirred at 90 °C for two hours while monitoring the reaction with TLC. The reaction was halted, and the solution was cooled to room temperature, filtered, and concentrated via rotary evaporation. Using flash silica gel chromatography (hexyl hydride–ethyl acetate = 30:1), the residue was purified to produce an orange solid weighing 851 mg with a 95% yield. The ^1^H and ^13^C NMR spectrum are displayed in the Supporting Materials (Figures S3 and S6).

### 3.3. Characterization

Purification was performed using the SepaBean machine automated flash chromatography system by Santai Technologies, Inc. (Pointe-Claire, QC, USA). ^1^H and ^13^C NMR spectra were obtained on a 500 MHz INOVA spectrometer (Varian, Palo Alto, CA, USA), using dimethyl sulfoxide-*d*_6_ or Chloroform-*d* as the solvent and tetramethylsilane as the standard. The samples were charged/discharged under 550/430 nm light irradiation. The optical power meter (CEL-NP2000, Beijing China Education Au-light Co., Ltd., Beijing, China) was applied to measuring the intensity of light. The X-Ray Diffraction (XRD) patterns were measured on a Rigaku SmartLab SE diffractometer (Tokyo, Japan). Differential Scanning Calorimetry (DSC) measurements were conducted on a DSC 214 (Netzsch, Selb, Germany).

### 3.4. UV-Vis Absorption Spectroscopy

Light Source Optimization: The sample was dissolved in dichloromethane at a 0.0100 mg/mL concentration. The UV–vis spectra of the *E*-isomers were first documented in the dark. Subsequently, the samples were irradiated with the specified wavelengths of the light source until no absorbance changes were observed, typically after 120 s or 2 h. After the irradiation, the UV–vis spectra were recorded as the photostationary state (PSS) at the specified wavelengths of the light sources. The light sources included 365 nm (50 mW cm^−2^), 395 nm (50 mW cm^−2^), 420 nm (50 mW cm^−2^), 430 nm (50 mW cm^−2^), 520 nm (20 mW cm^−2^), 530 nm (20 mW cm^−2^), 550 nm (20 mW cm^−2^), 590 nm (20 mW cm^−2^), and 610 nm (20 mW cm^−2^).

Time-Evolved Photoisomerization: The samples were irradiated using a 550 nm or 430 nm wavelength light source. During irradiation, the UV–vis absorption spectra were recorded at regular intervals, and the data obtained were normalized using software.

### 3.5. Lifetime Measurements

Samples of the 550 nm PSS liquid *Z* isomer were placed in a dark room at room temperature. The UV–vis absorption spectra of the samples were measured periodically until they returned to their original state, and the data collected were normalized using software.

### 3.6. DSC Measurements

The liquid *Z* isomer was obtained from the solid *E* isomer after 30 min of exposure to a 550 nm LED lamp. The samples were then moved to a DSC pan to measure their exothermic properties. Unless specified otherwise in the figure captions, the cooling and heating rates during the DSC tests were maintained at 10 °C/min.

### 3.7. Computational Studies

Density functional theory (DFT) was employed to calculate the single-point energies of the *Z* and *E* forms of *o*-4Clazo-C_8_. The geometry of each structure was optimized using the B3LYP method and the 6-31+G(d,p) basis set. A frequency analysis was then performed at the same theoretical level on each optimized structure to obtain their thermodynamic parameters. For more accurate computational outcomes, single point energies were calculated using the wB97XD/6-311++G(d,p) method for optimized geometries. All computations were performed using Gaussian 16 software, and the 3D molecular structures were visualized with CYLview 6.0.

### 3.8. Statistical Analysis

The data obtained in this study were used without preprocessing, unless otherwise noted. All data are presented as mean ± standard deviation, with measurements from at least three independent experiments. All experiments were performed with multiple replicates (triplicate to quintuplicate).

## 4. Conclusions

This study reports the successful design and synthesis of an azobenzene photothermal molecule, *o*-4ClAzo-C_8_, featuring a *tetra*-*ortho*-chlorinated azo structure as the head and an alkyl chain at the *para*-position as the tail. The *tetra*-*ortho*-chlorinated structure endows the photothermal molecule with the ability to effectively convert visible-light photon energy into chemical energy and store it in an N=N bond, before it is released as heat energy when needed. The photoisomerization properties of *o*-4ClAzo-C_8_ were researched through the UV–vis and ^1^H NMR spectra. Specifically, 64.9% of *Z*-isomer was obtained via charging under a 550 nm wavelength of light irradiation, and 87.8% of *E*-isomer was achieved via discharging at a 430 nm wavelength of light irradiation. The *o*-4ClAzo-C_8_ photothermal molecule demonstrated long-term energy storage with a half-life of approximately 11 days and maintained cyclic stability for 100 cycles, likely due to the steric hindrance effects of the *tetra*-*ortho*-substituted chlorine atom. The experimental energy storage density of the *Z*-isomer (13.50 kJ/mol) is lower than the theoretical calculation value (28.21 kJ/mol) due to the relatively low degree of *E*-to-*Z* isomerization. Remarkably, the *o*-4ClAzo-C_8_ photothermal molecule showed an irreversible photoinduced phase transition from an *E*-crystal to *Z*-liquid and a high degree of supercooling through XRD and DSC experiments, which may be due to the *para*-alkyl chain of molecules and the steric hindrance effect of the chlorine atoms. In the future, this azobenzene photothermal molecule may have potential applications in temperature buffering systems such as antifreeze protection for equipment and clothing used in polar or space explorations in extremely cold weather.

## Data Availability

The original contributions presented in the study are included in the article; further inquiries can be directed to the corresponding author.

## Supplementary Materials

The following supporting information can be downloaded at: https://www.mdpi.com/article/10.3390/molecules30112333/s1, Figure S1. The ^1^H NMR spectrum of *o*-4Clazo-OH molecule. Figure S2. The ^13^C NMR spectrum of *o*-4Clazo-OH molecule. Figure S3. The ^1^H NMR spectrum of *o*-4Clazo-C_8_ molecule in its initial state. Figure S4. The ^1^H NMR spectrum of *o*-4Clazo-C_8_ molecule in its 550 nm PSS. Figure S5. The ^1^H NMR spectrum of *o*-4Clazo-C_8_ molecule in its 430 nm PSS. Figure S6. The ^13^C NMR spectrum of *o*-4Clazo-C_8_ molecule. Figure S7. DSC curves of *o*-4ClAzo-C_8_ for (a) initial state (*E*-rich) along with heating (red), cooling (blue) and reheating (purple) segments.

## Abbreviations

The following abbreviations are used in this manuscript:

*o*-4ClAzo-OH	(*E*)-3,5-dichloro-4-((2,6-dichlorophenyl)diazenyl)phenol
*o*-4ClAzo-C_8_	(*E*)-1-(2,6-dichloro-4-(octyloxy)phenyl)-2-(2,6-dichlorophenyl)diazene
UV–vis	Ultraviolet–visible
XRD	X-ray diffraction
DSC	Differential scanning calorimetry
