# Supplementary material for: A tetra-ortho-Chlorinated Azobenzene Molecule for Visible-Light Photon Energy Conversion and Storage
Source: Molecules. 2025 May 27;30(11):2333. doi: 10.3390/molecules30112333 (PMC12155895; doi:10.3390/molecules30112333)
Supplement: Supplementary file 1 [file molecules-30-02333-s001.zip › molecules-3617394-supplementary.pdf]

Supplementary Material

A *tetra-ortho*-chlorinated azobenzene molecule for visible light photon energy conversion and storage

Shuxin Tang [1], Yating Zhang [1,3] Jun Xia [1], Jing Qi [1], Fan Tang [1], Fei Zhai [2,] and Liqi Dong [1,2,4,]**

1 Key Laboratory of Pollution Exposure and Health Intervention of Zhejiang Province, Interdisciplinary Research Academy, Zhejiang Shuren University, Hangzhou, 310021, PR China

2 Shandong Laboratory of Advanced Materials and Green Manufacturing at Yantai, Yantai Zhongke Research Institute of Advanced Materials and Green Chemical Engineering, Yantai 264006, P. R. China

3 College of Environment, Zhejiang University of Technology, 18 Chaowang RD, Hangzhou, 310014, P.R. China

4 Zhejiang Collaborative Innovation Center for Full-Process Monitoring and Green Governance of Emerging Contaminants, Hangzhou 310021, P. R. China

Corresponding Author: liqi_dong@tju.edu.cn (L.D.); zhaifei@amgm.ac.cn (F.Z.)

Table of Contents

1. Instruments and characterization 2

2. NMR spectrum 3

3. DSC curves 9

4. Equations 10

1. Instruments and characterization

The SepaBean machine automated flash chromatography system (Santai Technologies, Inc.) was used for Purification. ^{1}H and ^{13}C NMR spectra were obtained on a 500 MHz INOVA spectrometer (Varian, USA), using dimethyl sulfoxide-d_6 or Chloroform-*d* as the solvent and tetramethylsilane as the internal standard. The samples were charged/discharged under 550/430 nm light irradiation. The optical power meter (CEL-NP2000, Beijing China Education Au-light Co., Ltd.) was applied to measuring the intensity of light. The X-Ray Diffraction (XRD) patterns were measured on a Rigaku SmartLab SE diffractometer. Differential Scanning Calorimetry (DSC) measurements were conducted on a DSC 214 (Netzsch, Germany). All UV-vis absorption spectra were obtained using a UV-Vis spectrophotometer (V-750, JASCO, Japan).

2. NMR spectrum

o-4Clazo-OH

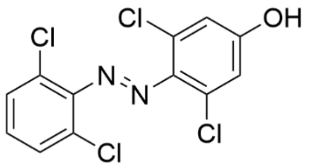

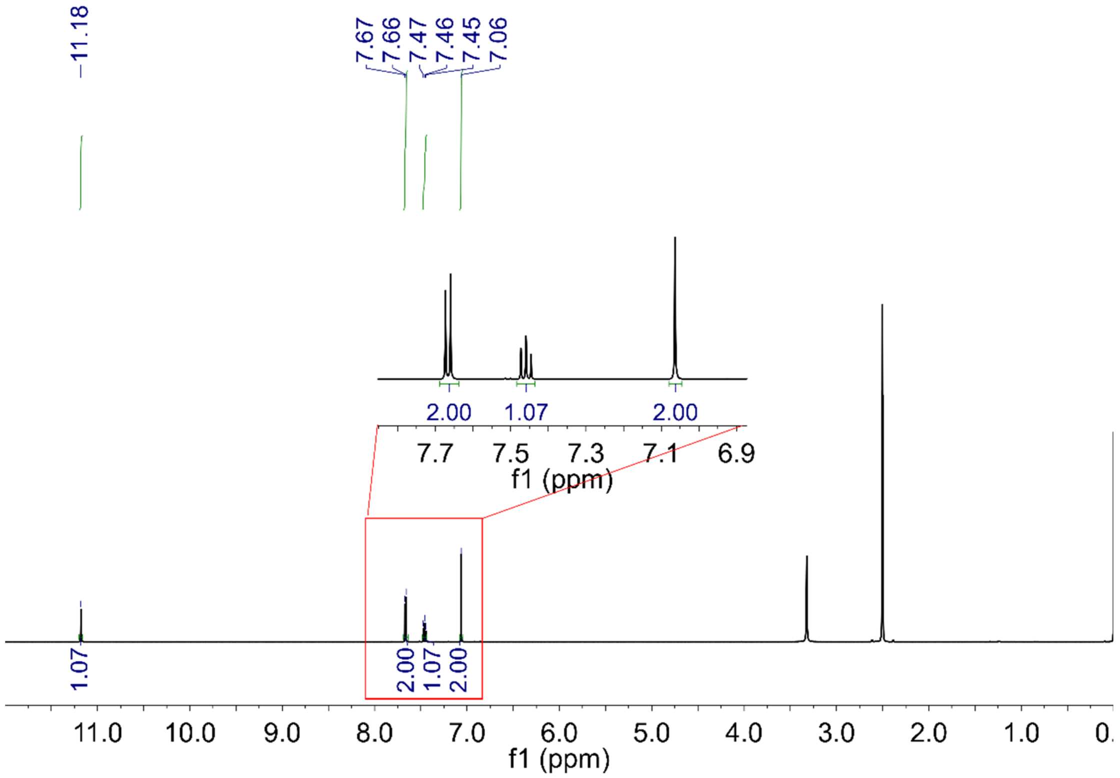

Figure S1. The ^{1}H NMR spectrum of *o*-4Clazo-OH molecule.

^{1}H NMR (400 MHz, DMSO-d_6) δ 11.18 (s, 1H), 7.67 (d, J = 8.2 Hz, 2H), 7.46 (t, J = 8.1 Hz, 1H), 7.06 (s, 2H).

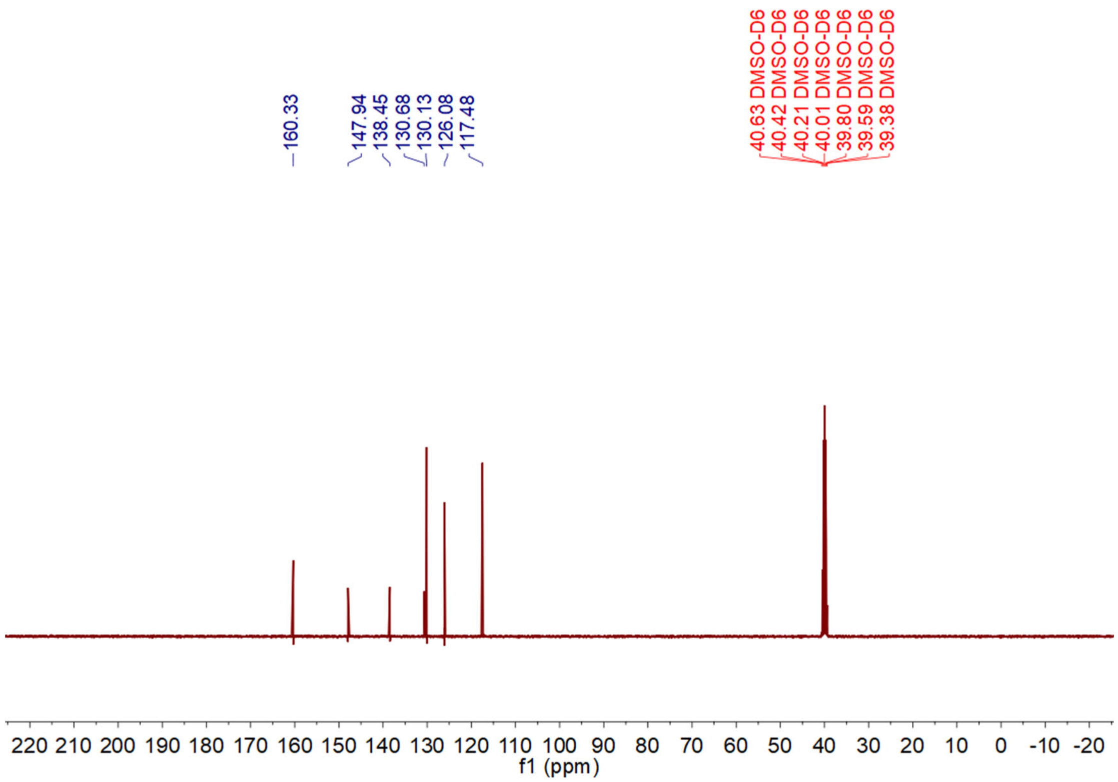

Figure S2. The ^{13}C NMR spectrum of *o*-4Clazo-OH molecule.

^{13}C NMR (101 MHz, DMSO-d_6) δ: 160.33, 147.94, 138.45, 130.68, 130.13, 126.08, 117.48.

o-4Clazo-C_8

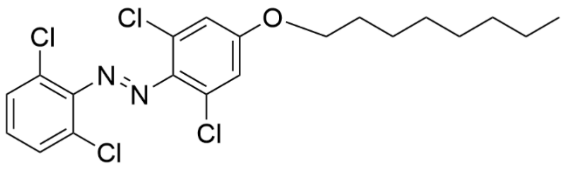

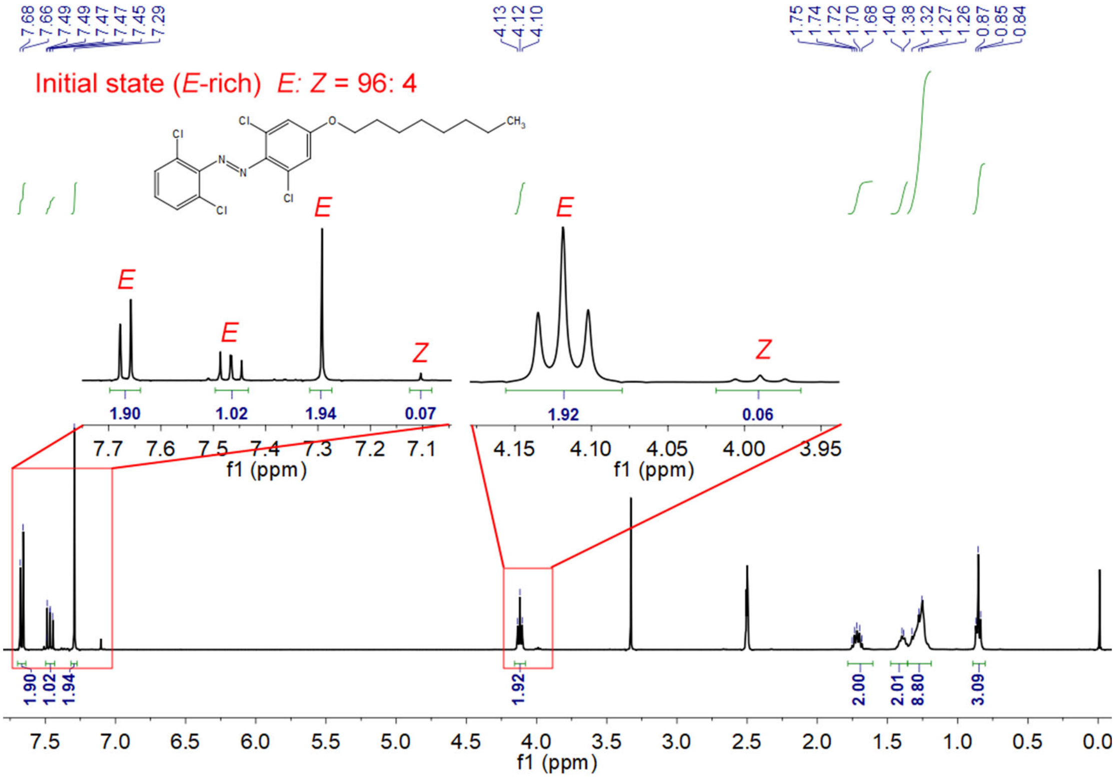

Figure S3. The ^{1}H NMR spectrum of *o*-4Clazo-C_8 molecule in its initial state.

^{1}H NMR (400 MHz, DMSO-d_6) δ 7.67 (d, J = 8.1 Hz, 2H), 7.47 (dd, J = 8.6, 7.7 Hz, 1H), 7.29 (s, 2H), 4.12 (t, J = 6.5 Hz, 2H), 1.75–1.68 (m, 2H), 1.39 (d, J = 4.7 Hz, 2H), 1.32–1.26 (m, 8H), 0.87–0.84 (m, 3H).

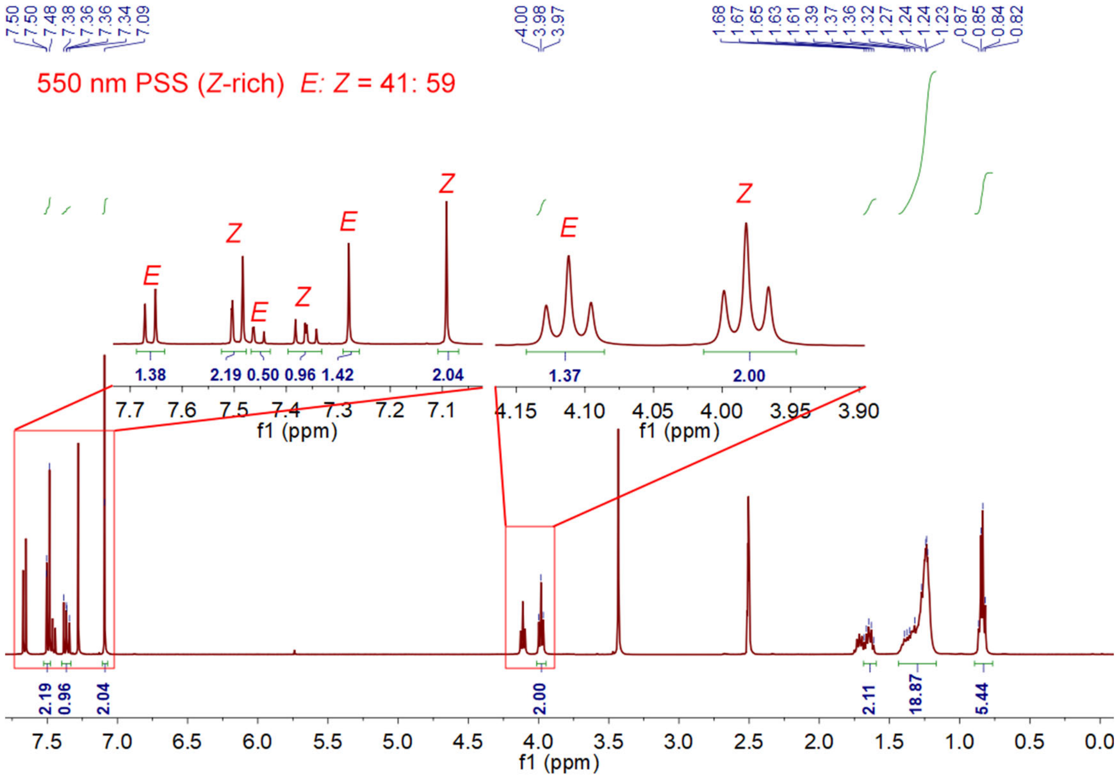

Figure S4. The ^{1}H NMR spectrum of *o*-4Clazo-C_8 molecule in its 550 nm PSS.

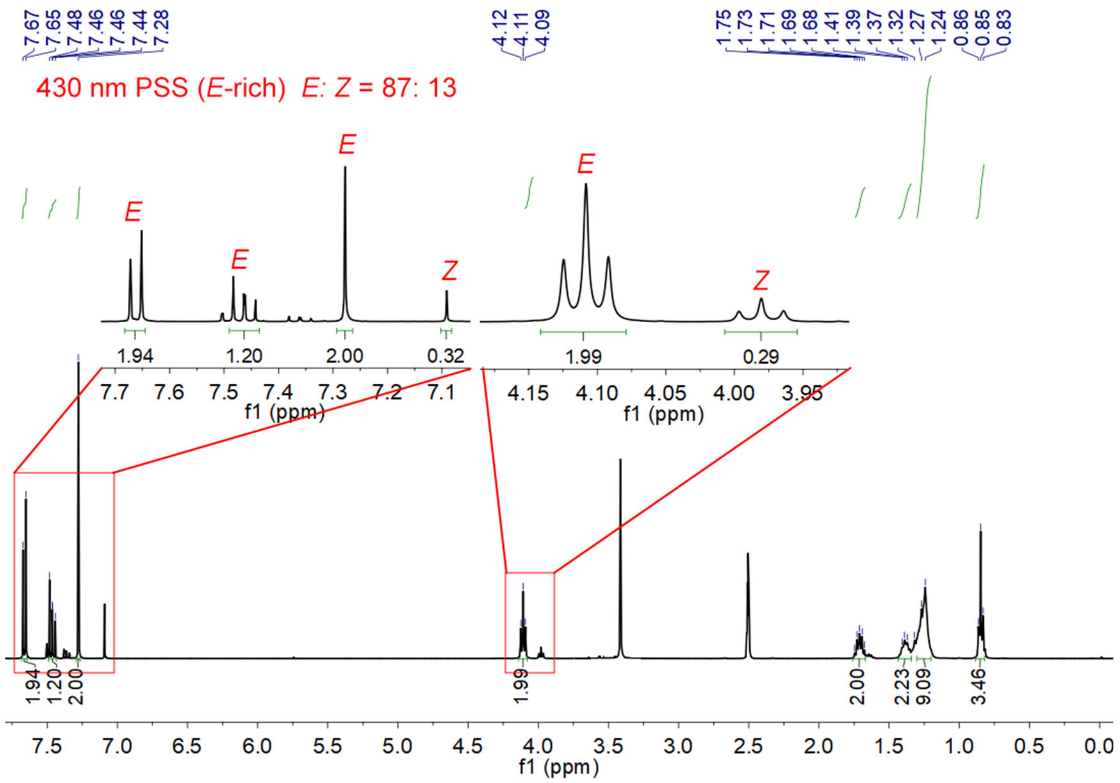

Figure S5. The ^{1}H NMR spectrum of *o*-4Clazo-C_8 molecule in its 430 nm PSS.

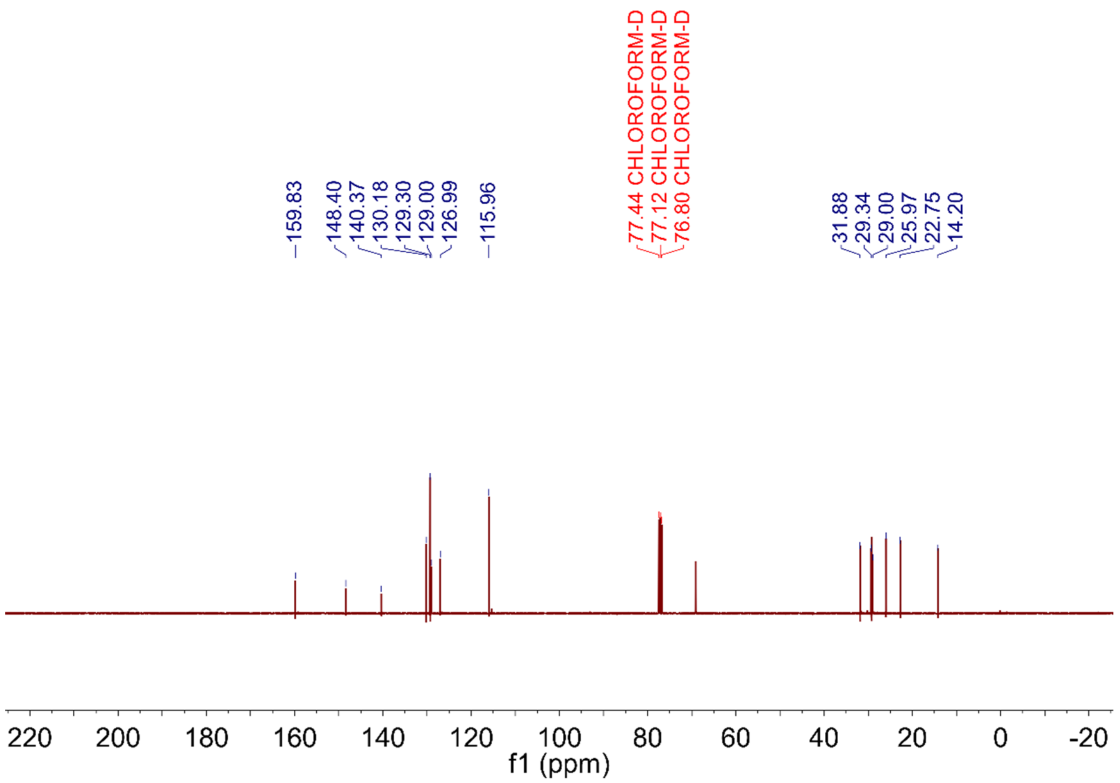

Figure S6. The ^{13}C NMR spectrum of *o*-4Clazo-C_8 molecule.

^{13}C NMR (101 MHz, Chloroform-*d*): δ 159.84, 148.41, 140.40, 130.19, 129.31, 127.00, 115.96, 31.88, 29.34, 29.00, 25.97, 22.75, 14.21.

3. DSC curves

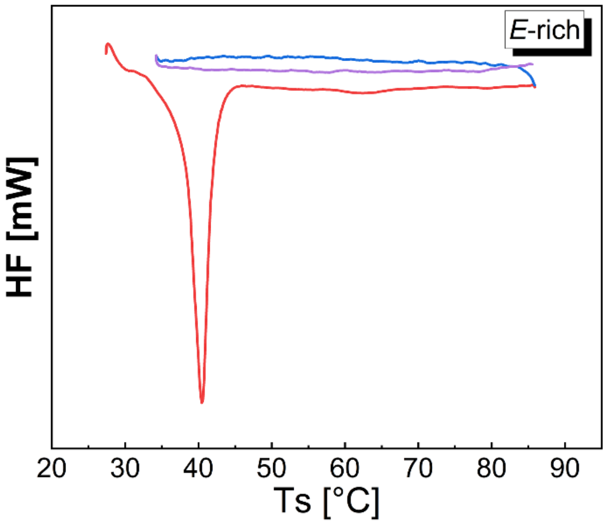

Figure S7. DSC curves of *o*-4ClAzo-C_8 for (a) initial state (*E*-rich) along with heating (red), cooling (blue) and reheating (purple) segments.

4. Equations

Equation S1. Fraction of isomers

According to the UV-Vis spectra, the fraction of isomers can be estimated as the percentage change of absorbance at the wavelength of the π-π* transition peak.

$$Z\ (\%) = \frac{A_t - A_E}{A_Z - A_E} \times 100\% \qquad \text{(S1)}$$

Where A_t is the absorbance intensity of samples at the irradiation time of "t", A_E is the absorbance intensity of 100% E-isomer. Az is the absorbance intensity of 100% Z-isomer. The initial state was assumed to be ~100% E-isomer, while the 610-nm PSS in dilute solutions was assumed to be ~100% Z-isomer.

Equation S2. Percentage of isomers

According to the ^{1}H NMR spectra, the fraction of isomers was calculated by the following Equation:

$$\mathrm{Z}\ (\%) = \frac{A_Z}{A_E + A_Z} \times 100\% \qquad \text{(S2)}$$

Where A_E and A_Z are the values of integral areas for the Z-isomer and E-isomer of azobenzene photoswitches in ^{1}H NMR spectra.

Equation S3. First-order kinetic constants (κ_{rev})

$$ln\left(\frac{A_\infty - A_t}{A_\infty - A_0}\right) = -\kappa_{rev} t \qquad \text{(S3)}$$

Where A_t is the absorption intensity of samples reversing for "*t*" time, A_0 is the absorption intensity of samples in Z-rich state irradiated by 550 nm light and A_∞ is the absorption intensity of samples in E-rich state irradiated by 430 nm light.

Equation S4. Energy storage lifetime ($\tau_{1/2}$)

$$\tau_{1/2} = \frac{\ln 2}{\kappa_{rev}} \qquad \text{(S4)}$$

Where κ_{rev} represents the first-order rate constant calculated by Equation S3.
